# A new score predicting the survival of patients with spinal cord compression from myeloma

**DOI:** 10.1186/1471-2407-12-425

**Published:** 2012-09-25

**Authors:** Sarah Douglas, Steven E Schild, Dirk Rades

**Affiliations:** 1 Department of Radiation Oncology, University of Lubeck, Ratzeburger Allee 160, D-23538, Lubeck, Germany; 2 Department of Radiation Oncology, Mayo Clinic Scottsdale, Scottsdale, AZ, USA

**Keywords:** Myeloma, Metastatic spinal cord compression, Radiotherapy, Survival prognosis, Scoring system

## Abstract

**Background:**

This study was performed to create and validate a scoring system for the survival of patients with malignant spinal cord compression (SCC) from myeloma.

**Methods:**

Of the entire cohort (N = 216), 108 patients were assigned to a test group and 108 patients to a validation group. In the test group, nine pre-treatment factors including age, gender, Eastern Cooperative Oncology Group performance status (ECOG-PS), number of involved vertebrae, ambulatory status prior to radiotherapy, other bone lesions, extraosseous lesions, interval from first diagnosis of myeloma to radiotherapy of SCC, and the time developing motor deficits were retrospectively analyzed.

**Results:**

On univariate analysis, improved survival was associated with ECOG-PS 1–2 (p = 0.006), being ambulatory (p = 0.005), and absence of other bone lesions (p = 0.019). On multivariate analysis, ECOG-PS (p = 0.036) and ambulatory status (p = 0.037) were significant; other bone lesions showed a strong trend (p = 0.06). These factors were included in the score. The score for each factor was determined by dividing the 12-month survival rate (in%) by 10. The total risk score was the sum of the three factor scores and ranged from 19 to 24 points. Three prognostic groups were designed with the following 12-month survival rates: 49% for 19–20 points, 74% for 21–23 points, and 93% for 24 points (p = 0.002). In the validation group, the 12-month survival rates were 51%, 80%, and 90%, respectively (p < 0.001).

**Conclusions:**

This score appears reproducible, because the 12-month survival rates of both the test and the validation group were very similar. This new survival score can help personalize the treatment of patients with SCC from myeloma and can be of benefit when counseling patients.

## Background

Myeloma is one of the most common malignant diseases leading to malignant spinal cord compression (SCC) [1,2]. In contrast to patients with SCC from a solid tumor, myeloma patients with SCC are usually not candidates for spinal surgery, because myeloma is an extraordinarily radiosensitive lesion [2]. Thus, patients with SCC from myeloma were exluded from the randomized trial of Patchell et al. that compared radiotherapy alone to radiotherapy plus upfront decompressive surgery in patients with SCC [3]. Therefore, radiotherapy alone is generally considered the standard treatment for SCC from myeloma [2]. Personalizing cancer care is one of the most important trends in oncology. In order to administer the best treatment regimen to the individual patient, it is mandatory to consider the patient's survival prognosis. Long term local control of SCC is dose dependent. As such, patients with a more favorable prognosis can benefit from longer-course radiotherapy (mostly 30 Gy in 10 fractions over 2 weeks) in terms of better local control, whereas patients with an unfavorable prognosis may only need to receive short-course radiotherapy with an overall treatment time of one week or less [4]. On the other hand, patients with an extraordinarily good survival prognosis appear to benefit from an escalation of the radiation dose beyond 30 Gy in 10 fractions [5]. These patients live long enough that local failure can be an issue. Therefore, it is important to be able to estimate the patient's survival prognosis. This could be achieved with the use of survival scores. Because different primary tumors have different biological behavior, it is important to have a specific survival score for each tumor entity associated with SCC [1,2]. Patients with SCC from myeloma must be considered unique, as they have the best survival prognosis of all patients developing SCC from a malignant disease [6]. In the study presented here, we create and validate a survival score for this group of patients.

## Results

In the test group, survival was associated with the Eastern Cooperative Oncology Group performance status (ECOG-PS) (p = 0.006), ambulatory status prior to radiotherapy (p = 0.005), and other bone lesions (p = 0.019) on univariate analysis (Table 1). In the corresponding multivariate analysis, ECOG-PS (p = 0.036) and ambulatory status prior to radiotherapy (p = 0.037) maintained significance (Table 2). The presence of other bone lesions showed a strong trend (p = 0.06). All three prognostic factors were included in the scoring system. The scores for each of these factors obtained from the 12-month survival rate are shown in Table 3.

**Table 1:** Test group: Univariate analysis of pre-treatment factors and the radiation regimen for survival

	**Survival at 6 months (%)**	**Survival at 12 months (%)**	**Median survival time (months)**	**p-value**
**Age**				
≤ 63 years	82	76	45	
≥ 64 years	88	73	57	0.92
**Gender**				
i Female	76	61	37	
Male	90	83	45	0.29
**ECOG Performance status**				
1-2	91	81	57	
3-4	73	60	14	**0.006**
**Number of involved vertebrae**				
1-2	83	73	70	
≥ 3	87	77	37	0.95
**Ambulatory status prior to radiotherapy**				
Not Ambulatory	70	56	14	
Ambulatory before RT	90	81	57	**0.005**
**Other bone lesions**				
No	98	84	not reached	
Yes	76	69	32	**0.019**
**Extraosseous lesions**				
No	86	75	45	
Yes	67	not available	not available	0.35
**Interval from cancer diagnosis to radiotherapy of MSCC**				
≤ 15 months	90	77	45	
> 15 months	80	73	32	0.19
**Time developing motor deficits**				
1-14 days	80	66	19	
> 14 days	89	81	57	0.27
**Radiation regimen**				
Short-course radiotherapy	81	72	45	
Longer-course radiotherapy	87	76	57	0.97

**Table 2:** Test group: Multivariate analysis of pre-treatment factors and the radiation regimen for survival

	**Risk ratio**	**95%-confidence interval**	**p-value**
**ECOG Performance status**	2.09	1.05 – 4.12	**0.036**
**Ambulatory status prior to radiotherapy**	2.14	1.05 – 4.24	**0.037**
**Other bone lesions**	1.97	0.96 – 4.37	0.06

**Table 3:** Test group: 12-month survival rates and corresponding scores

	**Survival at 12 months (%)**	**Score (points)**
**ECOG Performance status**		
1–2	81	8
3-4	60	6
**Ambulatory status prior to radiotherapy**		
Not Ambulatory	56	6
Ambulatory	81	8
**Other bone lesions**		
No	84	8
Yes	69	7

**Figure 1:**
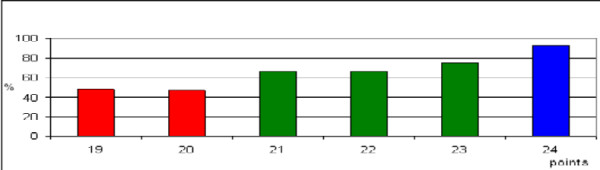
Test group: The total scores in relation to the 12-month survival rate (in%).

**Figure 2:**
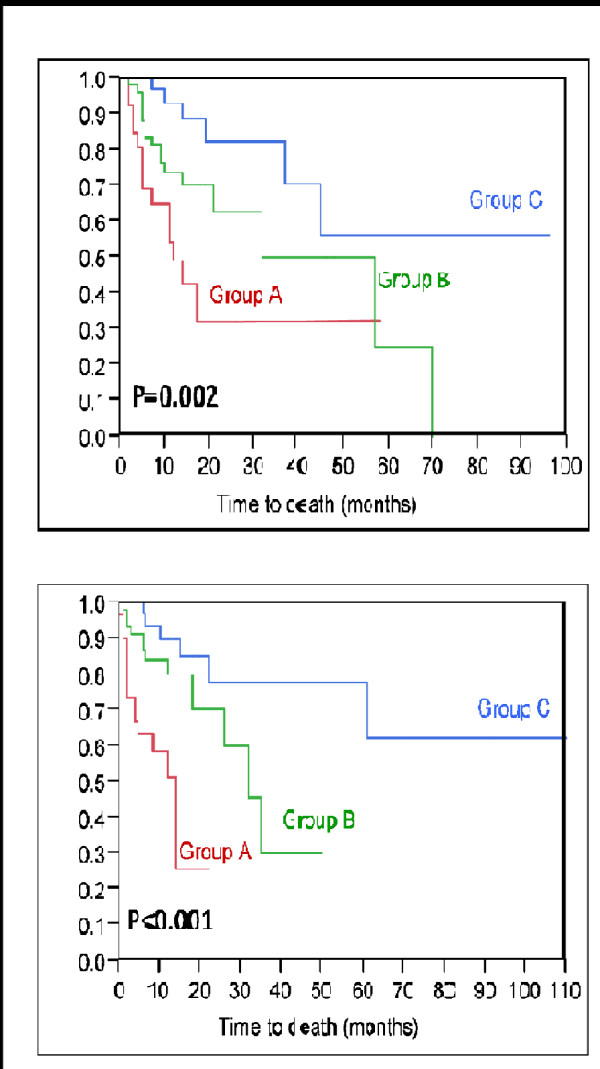
Kaplan-Meier curves for survival of the three score groups A (19–20 points), B (21–23 points), and C (24 points) from the test group (top) and from the validation group (bottom).

The addition of the three scores for each factor resulted in total scores from 19 to 24 points (Figure 1). According to the total scores, the patients of the test group were divided into three prognostic groups, 19–20 points (group A, n = 26), 21–23 points (group B, n = 49), and 24 points (group C, n = 33) (Figure 1). The 12-month survival rates were 49% for group A, 74% for group B, and 93% for group C (p = 0.002, Figure 2). The 12-month survival rates of the three prognostic groups A, B and C in the validation group were 51%, 80%, and 90%, respectively (p < 0.001, Figure 2). Thus, the corresponding survival rates of the test group and the validation group were very similar.

## Discussion

Personalization of cancer treatment has been increasingly emphasized in the literature. This is especially obvious when one reviews the new targeted agents that affect specific biochemical reactions and often have activity specific to certain mutations of a biologically active target molecule. Such an individualized approach should take into account the patient's survival time, particularly in a palliative situation such as malignant SCC. Therefore, survival scores that help the physician to tailor the treatment to the individual patients are important. Since the various tumors behave differently, it would be helpful to have a particular survival score for each tumor entity, at least for the most common diseases associated with SCC such as breast cancer, prostate cancer, non-small cell lung cancer, and myeloma [1,2]. In the present study, a survival score was designed particularly for patients with SCC from myeloma. The score included three prognostic factors, ECOG-PS, ambulatory status prior to radiotherapy, and other bone lesions. These factors found to be significantly associated with survival were also reported in our previous report on prognostic factors for SCC from myeloma [7]. In contrast to the test group of the present study, our previous report determined that extraosseous lesions were also significantly associated with survival. However, we decided to include only those prognostic factors found to be independent in the multivariate analysis of the test group in the present scoring system, because we felt that this would make the score more robust. This approach was supported by the fact that the 12-month survival rates of the validation group were similar to those of the test group.

Patients with SCC from myeloma have a better survival prognosis than patients with SCC from a solid tumor [1,2]. This is reflected by the fact that the worst prognostic group, group A, was the smallest group in this study. Patients of group C had a very good prognosis with 12-month survival rates of 93% in the test group and 90% in the validation group. According to a retrospective study, these patients appear to benefit from an escalation of the radiation dose beyond the world wide commonest regimen 30 Gy in 10 fractions in terms of better treatment outcomes [5]. This may also apply to patients of group B, whose 12-months survival rates were 74% and 80%, respectively. In contrast, group A patients who had the worst prognosis of the three groups, may be candidates for 30 Gy in 10 fractions or even a short-course regimen such as 20 Gy in 5 fractions. These suggestions should be interpreted with caution, because the data included in this survival score were retrospective in nature. Retrospective data always bear the risk of including hidden selection biases. However, it would be difficult and take many years to perform a prospective trial with an adequate number of patients with SCC from myeloma. The authors know of no plans to perform just such a prospective trial.

## Conclusions

This new survival score for patients with SCC from myeloma included three prognostic factors, ECOG-PS, ambulatory status prior to radiotherapy, and other bone lesions. Three prognostic groups were identified based on risk scores ranging from 19 to 24 points. The 12-month survival rates of each of the three prognostic groups in the validation group were similar to those of the test group. Therefore, this score can be considered reproducible. This information can contribute to personalization of the treatment for SCC from myeloma. Furthermore, the score can be helpful when counseling patients and relatives regarding prognosis and therapy.

## Methods

In this study, the data of 216 unselected patients being irradiated for SCC from myeloma between 1992 and 2011 were retrospectively analyzed. Because this study did not report on a clinical trial, and because the data were retrospective in nature and analyzed anonymously, approval by an ethic committee and informed consent from the patienst were not necessary. Patients included in this study received radiotherapy alone for SCC-related motor deficits of the lower extremities, did not have prior surgery or radiotherapy to the currently involved parts of the spinal cord, did have adequate diagnostic imaging with spinal CT or MRI was requested, and had received corticosteroid treatment during radiotherapy. Since the vast majority of the patients were already known as myeloma patients, a biopsy of the spinal lesions was not performed in most patients included in this study. The data were collected from the patients themselves, from their treating physicians, and from their files. Radiotherapy was performed with 6–10 MeV photon beams from a linear accelerator. The treatment volumes generally encompassed one normal vertebra above and below the involved parts of the spinal cord.

The 216 patients were alternately assigned to the test group (N = 108, uneven numbers) or the validation group (N = 108, even numbers). Patient characteristics of these groups are given in Table 4. In the test group, nine pre-treatment factors were investigated including age (≤63 vs.≥ years; median age: 63 years), gender, ECOG-PS (1–2 vs. 3–4), number of involved vertebrae (1–2 vs. ≥3), pre-radiotherapy ambulatory status (not ambulatory vs. ambulatory), other bone lesions prior to radiotherapy (no vs. yes), extraosseous lesions prior to radiotherapy (no vs. yes), interval between first diagnosis of myeloma and radiotherapy of SCC (≤15 vs. >15 months), and time of developing motor deficits prior to radiotherapy (1–7 vs. >7 days). In the entire cohort, the 12-month survival rate was 83% in patients who could walk without aid prior to radiotherapy and 80% in those patients who could walk with aid (p = 0.92). Because the 12-month survival rates were very similar, these two groups were combined to the group “ambulatory”.

**Table 4:** Patient characteristics of the test group and the validation group. The p-values were obtained from the Chi-square test

	**Test group**	**Validation group**	**p-value**
	**n patients (%)**	**n patients (%)**	
**Age**			
≤ 63 years	57 (53)	54 (50)	
≥ 64 years	51 (47)	54 (50)	0.84
**Gender**			
Female	38 (35)	42 (39)	
Male	70 (65)	66 (71)	0.81
**ECOG Performance status**			
1-2	75 (69)	70 (65)	
3-4	33 (31)	38 (35)	0.73
**Number of involved vertebrae**			
1-2	48 (44)	50 (46)	
≥ 3	60 (56)	58 (54)	0.91
**Ambulatory status prior to radiotherapy**			
Not Ambulatory	27 (25)	32 (30)	
Ambulatory before RT	81 (75)	76 (70)	0.75
**Other bone lesions**			
No	45 (42)	43 (40)	
Yes	63 (58)	65 (60)	0.92
**Extraosseous lesions**			
No	105 (97)	102 (94)	
Yes	3 (3)	6 (6)	0.89
**Interval from cancer diagnosis to radiotherapy of MSCC**			
≤ 15 months	58 (54)	59 (55)	
> 15 months	50 (46)	49 (45)	0.95
**Time developing motor deficits**			
1-14 days	46 (43)	50 (46)	
> 14 days	62 (57)	58 (54)	0.78
**Radiation regimen**			
Short-course radiotherapy	37 (34)	38 (35)	
Longer-course radiotherapy	71 (66)	70 (65)	0.96

In addition to the pre-treatment factors, the potential impact of the radiation regimen (short-course radiotherapy with 1x8 Gy or 5x4 Gy over 1 week vs. longer-course radiotherapy with 10x3 Gy over 2 weeks, 14-15x2.5 Gy over 3 weeks, or 20x2 Gy over 4 weeks) has been investigated. Since other prognostic factors of multiple myeloma such as beta-2 myoglobulin and serum creatinine were not available in most patients being treated for SCC, an oncologic emergency situation, these factors were not included in the present study.

The univariate analysis was performed with the Kaplan-Meier-method and the log-rank test [8]. The significant prognostic factors (p < 0.05) were included in a multivariate analysis performed with the Cox proportion hazards model. The prognostic factors that were significant in the multivariate analysis of the test group (p < 0.05) or showed a strong trend (p≤0.06) were included in the survival score. The score for each significant prognostic factor was determined by dividing the 12-month survival rate (in%) by 10 according to our previous survival score that included many different primary tumor types [9]. The total score represented the sum of the scores for each factor.

## Abbreviations

ECOG-PS: Eastern Cooperative Oncology Group performance score; Gy: Gray; MeV: Mega electron volts; SCC: Spinal cord compression.

## Competing interests

The authors declare that they have no competing interests.

## Authors' contributions

SD, SES and DR participated in the design of the study. SES performed the statistical analyses. SD and DR provided study material. All authors were involved in manuscript writing. They read and approved the final manuscript.

## Pre-publication history

The pre-publication history for this paper can be accessed here:

http://www.biomedcentral.com/1471-2407/12/425/prepub
